# Association between gut microbiota and diabetic nephropathy: a two-sample Mendelian randomization study

**DOI:** 10.3389/fendo.2024.1361440

**Published:** 2024-07-04

**Authors:** Zhitao Ye, Tikyeung So, Tianyou Zhang, Xia Gao

**Affiliations:** ^1^ Department of Nephrology, Guangzhou Women and Children’s Medical Center, Guangzhou Medical University, Guangzhou, China; ^2^ Guanghua School of Stomatology, Sun Yat-sen University, Guangzhou, China; ^3^ Department of Urology, The Sixth Affiliated Hospital, Sun Yat-sen University, Guangzhou, China

**Keywords:** diabetic nephropathy, gut microbiota, mendelian randomization, single nucleotide polymorphism, genome-wide association studies

## Abstract

**Background:**

To clarify the causal relationship between gut microbiota and diabetic nephropathy (DN), we employed Mendelian randomization (MR). Despite a strong correlation observed, establishing causality is still unclear. By utilizing MR, we aimed to investigate this relationship further and shed light on the potential causal effect of gut microbiota on DN.

**Methods:**

Genetic instrumental variables for gut microbiota were obtained from a GWAS with 18340 participants. DN summary statistics (1032 cases, 451248 controls) were sourced from a separate GWAS. The primary analysis used the inverse-variance weighted (IVW) method. Reverse MR analysis was conducted to explore reverse causation. Rigorous sensitivity analyses were performed to ensure the resilience and reliability of the study’s findings.

**Results:**

We found two bacterial traits associated with an increased risk of DN: genus LachnospiraceaeUCG008 (OR: 1.4210; 95% CI: 1.0450, 1.9322; p = 0.0250) and genus Terrisporobacter (OR: 1.9716; 95% CI: 1.2040, 3.2285; p = 0.0070). Additionally, phylum Proteobacteria (OR: 0.4394; 95% CI: 0.2721, 0.7096; p = 0.0008) and genus Dialister (OR: 0.4841; 95% CI: 0.3171, 0.7390; p = 0.0008) were protective against DN. Sensitivity analyses consistently supported these results. In the reverse MR analysis, no statistically significant associations were observed between DN and these four bacterial traits.

**Conclusions:**

Our analyses confirmed a potential causal relationship between certain gut microbiota taxa and the risk of DN. However, additional studies are required to elucidate the underlying mechanisms through which gut microbiota influences the development of DN.

## Background

Diabetes is a globally prevalent disease with high rates of incidence and mortality, affecting over 10.5% of the population in 2021 (1). Diabetic nephropathy (DN) is the most common complication among individuals with diabetes, impacting around 40% of those affected (2). DN refers to kidney damage caused by diabetes and its prevalence is on the rise due to increasing rates of diabetes and obesity (3). Unfortunately, effective treatment options for DN are limited. Lifestyle interventions and medication management for blood sugar, blood pressure, and lipid control are currently the main strategies for managing the condition (4, 5).

The pathogenesis of DN involves complex interactions between genetic and environmental factors that are not yet fully understood (6). Recent studies have indicated that the gut microbiota may play a significant role in the development of DN (7). The concept of the gut-kidney axis, introduced in 2011, highlighted the influence of the intestinal tract on chronic kidney disease (CKD). Researchers have observed specific microbial changes in DN patients, such as a reduction in Roseburia intestinalis and an increase in Bacteroides stercoris (8). In a comprehensive analysis of fecal samples, enriched genera like Klebsiella, Citrobacter, and Escherichia coli were associated with DN, while Roseburia was found to be decreased (9). Variations in the abundance and diversity of gut microbiota have also been observed across different stages of DN, with specific genera like Shigella, Oscillatoria, and Hemophilus potentially serving as microbial biomarkers for diagnosing DN (10). These findings provide valuable insights into the relationship between the gut microbiota and DN, opening doors for future research and potential therapeutic interventions.

The intricate relationship between the gut microbiota and DN has been acknowledged, but establishing a definitive causal link remains challenging. Mendelian randomization (MR) analysis has emerged as a robust approach to address this challenge. MR helps to avoid confounding factors by utilizing genetic variants as instrumental variables (IVs). These genetic variants are randomly allocated during meiosis and are assumed to be independent of confounders. By leveraging these genetic variants, MR provides an inherent control for confounding, similar to a randomized controlled trial. Therefore, the associations reported in our study are less likely to be influenced by confounding bias or reverse causation (11).

To unravel the complex interplay between the gut microbiota and DN and identify specific pathogenic bacterial groups, a two-sample MR analysis was conducted using summary data from genome-wide association studies (GWAS). This approach provides more reliable and nuanced insights by using genetic variation as a natural experiment, overcoming limitations associated with observational studies and establishing a stronger foundation for inferring causality. This research represents a significant advancement in understanding the complexities of the gut microbiota’s role in DN and contributes valuable knowledge to the field of diabetes-related complications, paving the way for targeted interventions.

## Methods

### Data sources

**Figure 1:**
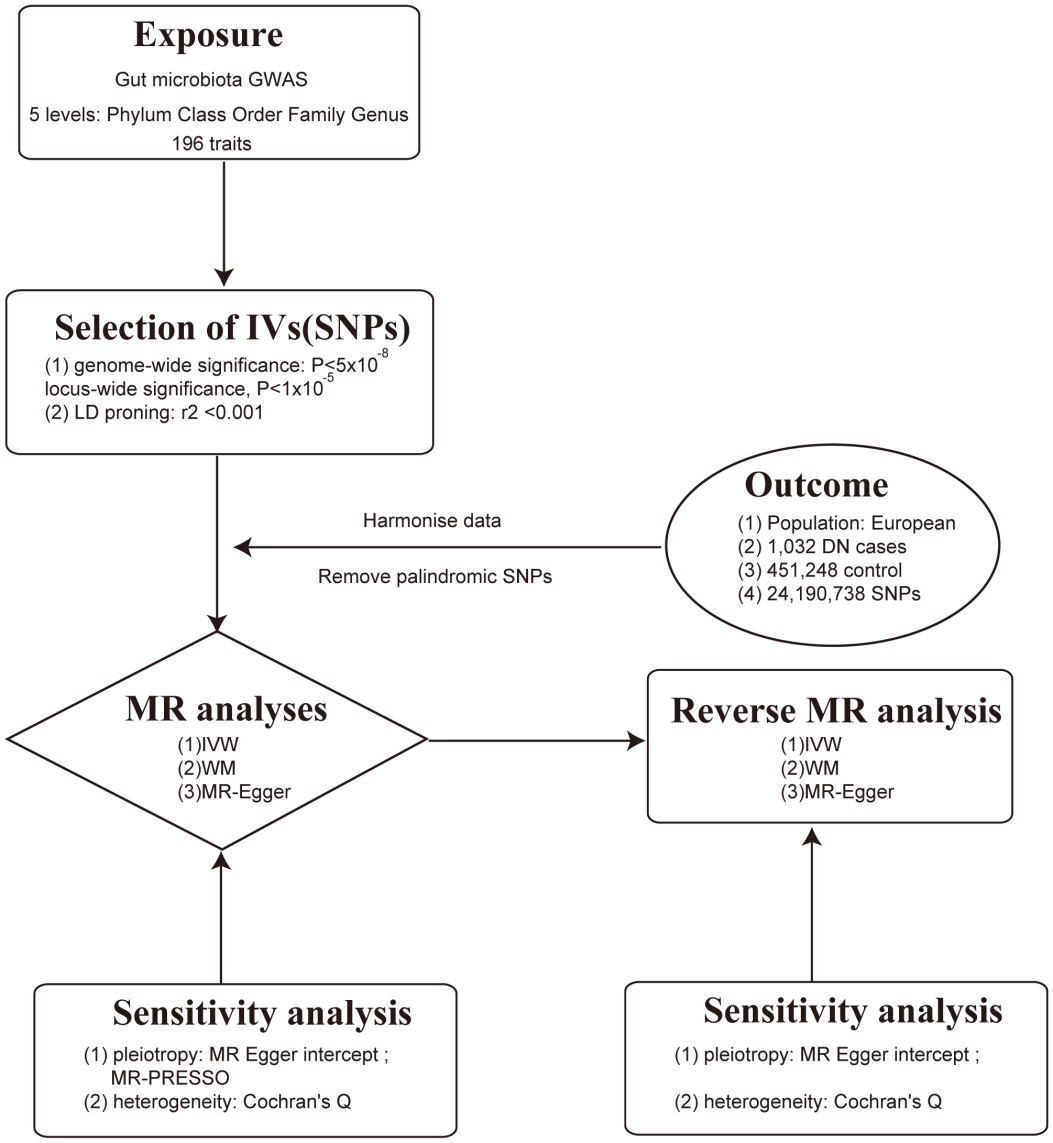
Mendelian randomization flowchart for gut microbiota and diabetic nephropathy.

This study employed a comprehensive genome-wide meta-analysis conducted by the MiBioGen consortium, involving 18340 individuals from mixed ancestry, to investigate the genetic variations associated with gut microbiota composition (12). The genetic summary statistics specific to DN were derived from a GWAS with 1032 cases and 451248 controls of European ancestry (13, 14). Rigorous adjustments were made for various factors, including age, sex, technical covariates, and genetic principal components. After excluding unidentified gut microbes, the analysis focused on 196 taxa out of the initially considered 211 taxa. The study’s design, outlined in **Figure 1**, establishes a flow diagram for exploring the potential causal link between gut microbiota composition and DN. Ethical review and approval was not required for the study on human participants in accordance with the local legislation and institutional requirements. Ethical approvals or participant consents had been obtained from original GWAS studies. Detailed information on data sources and characteristics can be found in **Table 1**.

**Table 1:** Details of the datasets used in the analyses.

Exposure or outcome	Sample size	Population	Data source	PMID
Human gut microbiome	18,340	Mixed	https://mibiogen.gcc.rug.nl/	33462485
Diabetic nephropathy	452,280	European	https://gwas.mrcieu.ac.uk/datasets/ebi-a-GCST90018832/	34594039

### Instrumental variables

**Figure 2:**
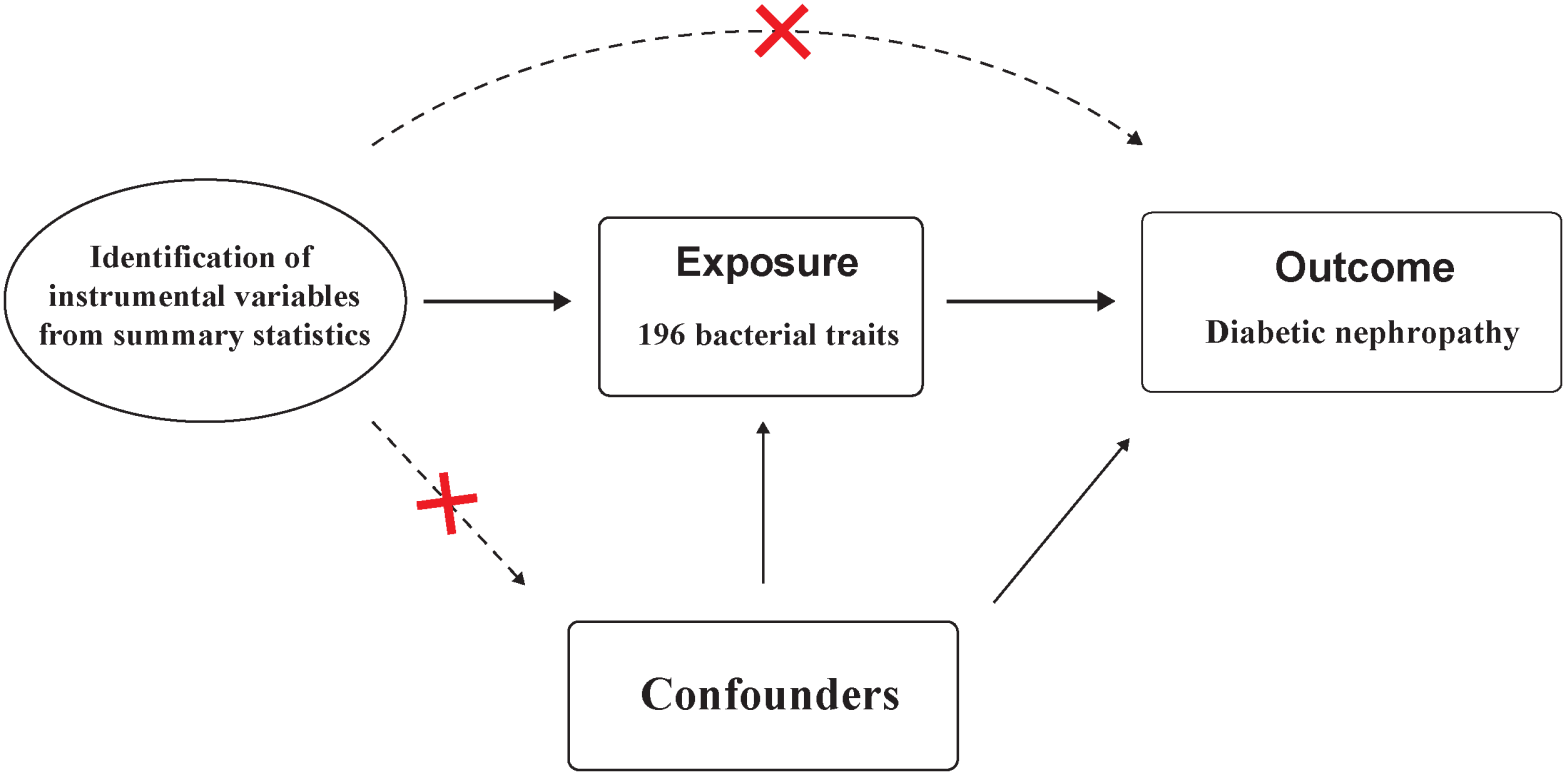
Assumptions in MR studies: a brief overview.

Instrumental variables (IVs) used for MR analysis should satisfy three key assumptions: 1) IVs are strongly associated with the exposure; 2) IVs should not associated with confounding factors; and 3) IVs affect the outcome solely through its impact on the exposure.(**Figure 2**) These assumptions form the foundation of MR analysis, which utilizes genetic variants as tools to infer causal connections between exposures and outcomes (15).

The selection of IVs for investigating DN involved a rigorous process. A stringent p-value threshold (p < 1.0 x 10^-5^) was applied, ensuring the IVs were highly associated with the exposure. Independence among IVs was established using an LD threshold (r2 < 0.001) and a clumping window of 10,000 kb, implemented with the “TwoSampleMR” package and 1000 Genomes EUR data. Instrument strength was assessed using F-statistics, with the following equation: F = β^2^/se^2^, with values above 10 indicating minimal weak instrumental bias (16). In reverse MR analysis, a more stringent p-value threshold (p < 5 x 10^-8^) was applied for selecting IVs associated with DN. These stringent criteria and advanced statistical measures were employed to identify reliable IVs and enhance the robustness of MR analyses when investigating causal relationships with DN. Proxy SNPs is to represent or substitute for the variations associated with the SNP of interest, aiding researchers in assessing the causal relationship between that SNP and specific traits (17). By setting the r2 threshold to 0.8, we ensure a high degree of association between the proxy SNP and the target SNP. Palindromic SNP refers to a single nucleotide polymorphism that exhibits a palindrome structure, where the nucleotide sequence reads the same forward and backward (17). For our study, palindromic SNPs with intermediate effect allele frequencies (effect allele frequency between 0.3 and 0.7) or SNPs with incompatible alleles were excluded from the analysis.

### Statistical analysis

To assess the potential causal connection between gut microbiota and DN, we employed a variety of analytical methods, including the IVW method, weighted median method, MR-Egger regression, and MR-PRESSO test. The IVW method which combined causal estimates of individual IVs was chosen as the primary analysis. Sensitivity analyses were conducted to ensure the reliability of our findings (18). MR-Egger regression was utilized to detect potential horizontal pleiotropy, assuming an instrument strength independent of direct effect (InSIDE). The intercept of MR-Egger reflected degree of horizontal pleiotropy and a p-value greater than 0.05 indicated no significant horizontal pleiotropy was presented (18). The MR-PRESSO test was employed to detect horizontal pleiotropic outliers and correct any potential distortions (19). Heterogeneity was assessed by Cochran’s Q value. Reverse MR analysis was selectively utilized to investigate the influence of DN on gut microbiota. Scatter plot and forest plots were drawn to visualize the causal effect of each individual IVs. All MR analyses were conducted using R version 4.3.0 with the “TwoSampleMR” package, ensuring consistency and reliability throughout the analytical procedures.

## Results

### Main results of the 196 bacterial traits with the risk of DN

**Figure 3:**
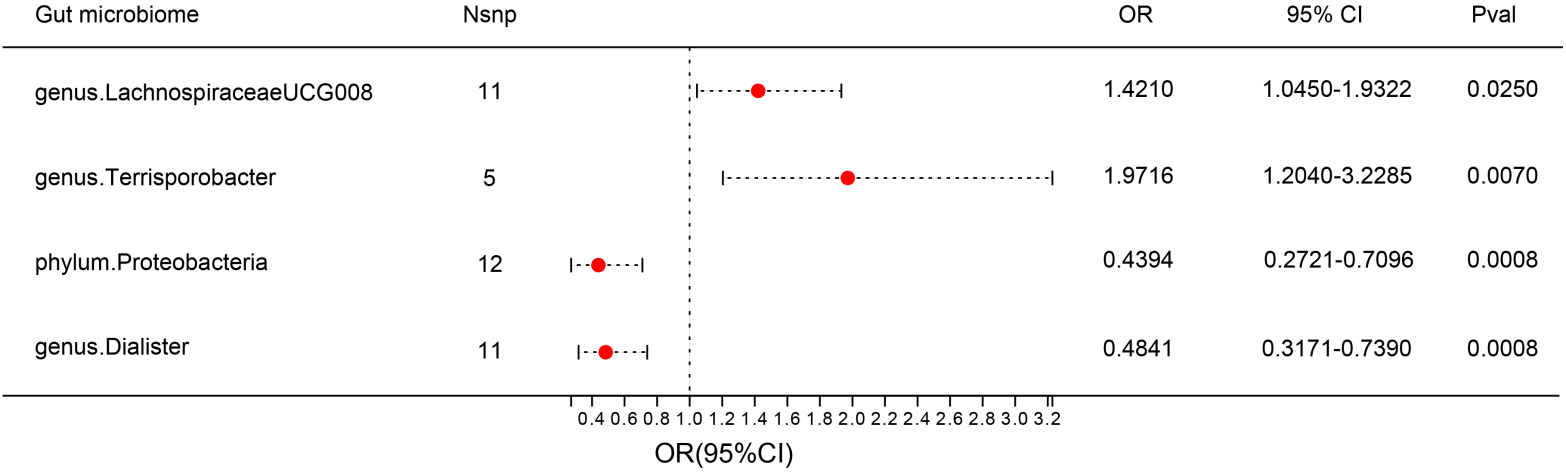
Forest plot: associations of genetically determined bacterial traits with diabetic nephropathy risk.

**Figure 4:**
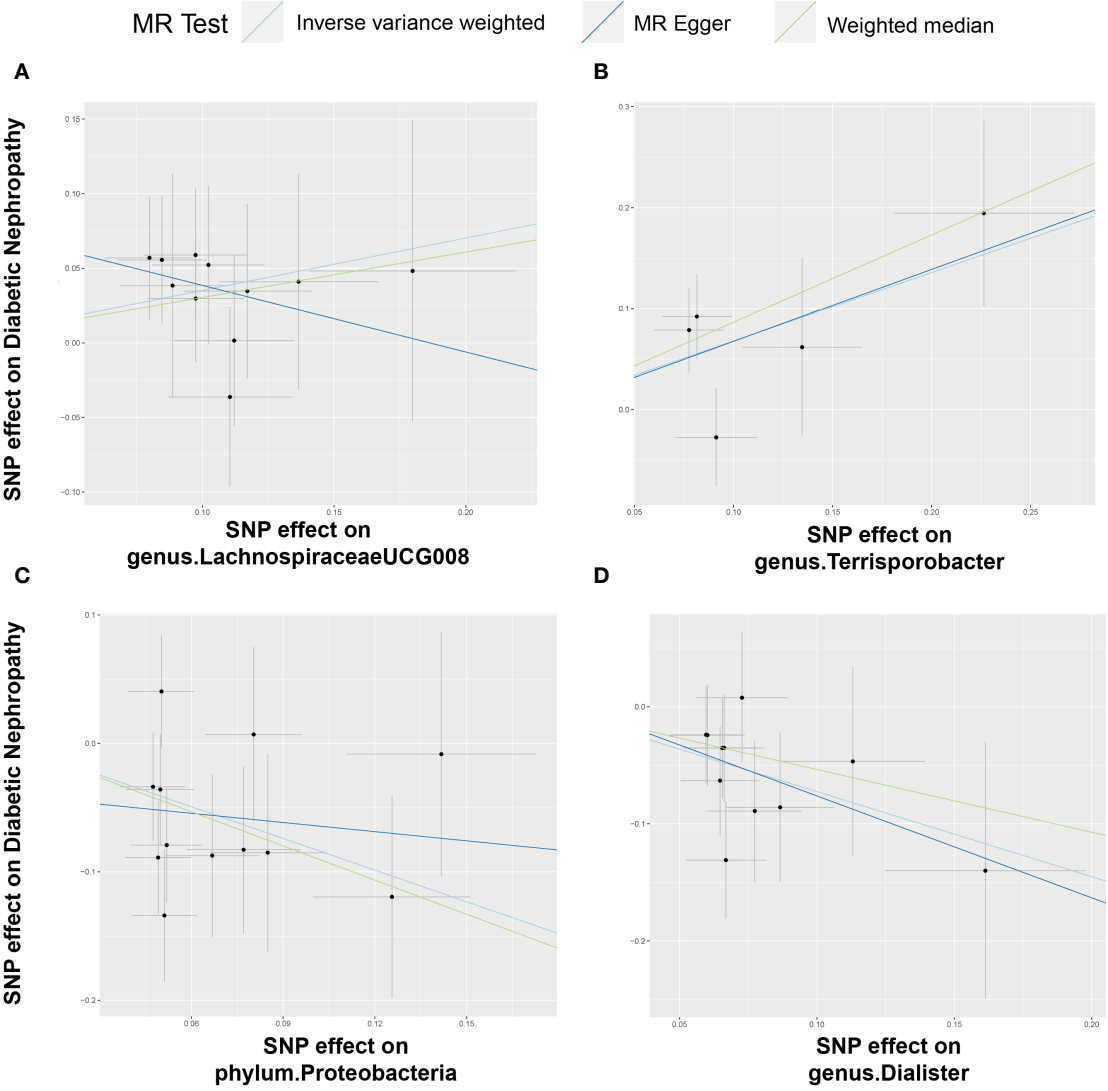
Scatter plot illustrating the associations between four bacterial traits on the risk of DN. **(A)** Causal effect of genus.LachnospiraceaeUCG008 on DN; **(B–D)** Causal effect of 3 other bacterial traits on DN.

F-statistics of SNPs associated with 196 bacterial traits ranged from 14.5893 to 88.4300 (20). **Supplementary Tables 1** provides information of IVs associated with gut microbiota. **Supplementary Table 2** showed association between these IVs and DN. **Supplementary Table 3** presented MR results for all traits and their association with DN risk, revealing four traits with suggestive associations using the IVW method. IVW method identified four bacterial traits associated with DN risk (**Figures 3**, **4** and **Supplementary Table 4**). The genetically predicted relative abundance of four taxa, namely LachnospiraceaeUCG008, Terrisporobacter, Dialister, and phylum Proteobacteria was assessed.

Employing the IVW method, a positive correlation emerged between genetically predicted LachnospiraceaeUCG008 and DN risk (OR = 1.4210; 95% CI: 1.0450, 1.9322; P = 0.0250). Both MR-PRESSO and MR-Egger analyses detected no outliers or directional pleiotropic effects. However, despite these findings, both MR Egger and weighted median analyses did not substantiate a causal link between LachnospiraceaeUCG008 and DN. Similarly, the genus Terrisporobacter exhibited a positive association with DN using the IVW method (OR: 1.9716; 95% CI: 1.2040, 3.2285; P = 0.0070). MR-PRESSO and MR-Egger analyses revealed no outliers or horizontal pleiotropy, yet only the weighted median analysis supported a causal positive relationship (OR: 2.3745; 95% CI: 1.2828, 4.3953; P = 0.0059). Both MR Egger and weighted median analyses did not substantiate a causal link between LachnospiraceaeUCG008 and DN (**Supplementary Table 5**, **Supplementary Table6**).

Contrary to previous findings, the genus Dialister exhibited a negative association with the risk of DN when assessed using the IVW method (OR = 0.4841; 95% CI: 0.3171, 0.7390; P = 0.0008). MR-Egger and MR-PRESSO tests indicating no evidence of directional pleiotropy or pleiotropic effects. Direction of result of MR Egger and weighted median analyses consistent with IVW method. Similarly, the phylum Proteobacteria showed a negative association with DN risk according to the IVW method (OR = 0.4394; 95% CI: 0.2721, 0.7096; P = 0.0008). No indications of directional pleiotropy or outliers were observed in MR-Egger or MR-PRESSO tests (**Supplementary Table 5**, **Supplementary Table 6**). Result of weighted median method support the finding of main analysis. Additional analyses presented in **Supplementary Table 7** demonstrated no significant heterogeneity among gut microbiota IVs.

### The result of reverse MR analysis

The associations between four bacterial traits and DN were investigated through reverse MR analyses. Utilizing the inverse variance-weighted (IVW) method, no statistically significant links were observed: genus LachnospiraceaeUCG008 (OR: 0.9901; 95% CI: 0.9481, 1.0338; P = 0.6508), phylum Proteobacteria (OR: 1.0208; 95% CI: 0.9936, 1.0487; P = 0.1354), genus Dialister (OR: 1.0161; 95% CI: 0.9829, 1.0505; P = 0.3457), and genus Terrisporobacter (OR: 1.0131; 95% CI: 0.9687, 1.0596; P = 0.5698). Neither MR-Egger nor weighted median analyses provided substantial support for a causal relationship involving any of the investigated traits. Sensitivity analyses were conducted to assess the stability of these findings, reinforcing the absence of a discernible causal link between the examined bacterial traits and the risk of developing DN (**Supplementary Table 8**, **Supplementary Table 9**).

## Discussion

The current research primarily focuses on identifying differences in the abundance of certain bacterial genera between disease groups and healthy individuals, but direct causal relationships between gut microbiota and diseases are still lacking. Our study employed Mendelian randomization to demonstrate that LachnospiraceaeUCG008 and Phylum Proteobacteria are risk factors for DN, while Dialister and Terrisporobacter are protective factors.

LachnospiraceaeUCG008, a member of the Lachnospiraceae family, is known for fermenting plant-derived polysaccharides into short-chain fatty acids and ethanol. Diabetes plays a crucial role in initiating CKD and influencing the progression of renal failure and cardiovascular complications (21). Disrupted glucose metabolism is significantly associated with the bacterial family Lachnospiraceae, which may explain its proliferation. Another study found a positive correlation between the presence of LachnospiraceaeUCG008 and the secretion of inflammatory factors (IL-6, HS-CRP, and TNF-α), suggesting a potentially harmful role in inflammation management (22, 23).

Phylum Proteobacteria is positively associated with DN in this study. Previous studies have investigated that the overgrowth of Proteobacteria has been associated with metabolic syndrome and inflammation (24). The main characteristics of type 2 DM are hyperglycemia and insulin resistance, and the chronic inflammation state exists throughout the whole progression of type 2 DM (25, 26). The phylum Proteobacteria may be one of the variables influencing DN. A study has discovered that patients with DN exhibit a greater diversity of gut bacteria compared to a control group (27). This research also pointed out an elevated presence of microbes from the Proteobacteria phylum in individuals with DN. Furthermore, recent findings have shown that an imbalance in the intestinal microbiome can lead to a malfunction in the intestinal barrier and bacterial translocation (28). This chain of events can ultimately induce a state of continuous systemic inflammation in patients suffering from CKD and finally evolve into DN.

Currently, the impact of Dialister and Terrisporobacter on serum lipid concentrations need to be better understood due to insufficient research. We noticed that both Dialister were relative to glucose metabolism. At the genus/species levels, a decreased abundance of Dialister, was observed in the disease groups in at least two studies, which is consistent with our findings (29, 30). Diego A. Esquivel-Hernández et al. pointed out that Terrisporobacter played an important role in diabetes mellitus. However, further investigations into the Terrisporobacter genus are required to elucidate these observed correlations’ mechanisms and causal relationships. For the first time, our findings confirm the protective potential of Dialister and Terrisporobacter in humans, implying that this taxon could be a novel biomarker. However, the underlying mechanism should be further explored.

The human gut microbiome is a multifaceted ecosystem within the body. It hosts not only bacteria but also viruses, fungi, and archaea. Research has uncovered encouraging links between certain fungal species and kidney health, specifically pointing to their association with the estimated glomerular filtration rate (eGFR), which may imply a beneficial role for these fungi in kidney function. Furthermore, connections have been observed between specific fungal genera, including Septoria, Nakaseomyces, and Saccharomyces, and blood pressure regulation, notably in relation to diastolic blood pressure. These findings suggest that these fungi could be influencing the mechanisms that control blood pressure (31, 32). Research has also documented significant interactions between bacteria and viruses within the gut, which are linked with both health and disease states. Evidence from studies indicates that Type 2 Diabetes (T2D) and DN are marked by alterations in the diversity and taxonomic makeup of gut viruses when juxtaposed with healthy individuals. A notable reduction in viral diversity, shifts in particular viral species, a decrease in various viral functions, and interruptions in the interplay between viruses and bacteria all point to a crucial role for the gut virome in contributing to the onset of T2D and DN (33).

Several limitations should be acknowledged in this study. Firstly, the majority of participants in the GWAS were of European descent, limiting the generalizability of the findings to other racial or ethnic populations. Secondly, the IV selection process included a less stringent p-value threshold (p< 1.0 × 10^-5^) to obtain adequate IVs. However, this approach may introduce false positives or miss significant genetic variants associated with the bacterial traits, so using a traditional genome-wide significance level (p< 5 × 10^-8^) for IVs selection would enhance the reliability of the results. Lastly, the study did not explore DN’s subtypes or specific characteristics. DN is a complex and heterogeneous condition, and investigating associations with bacterial traits in different DN subtypes or clinical features is crucial. These limitations underscore the need for future research with larger GWAS datasource.

## Conclusion

Through MR studies, we demonstrated that LachnospiraceaeUCG008 and Phylum Proteobacteria are risk factors for DN, while Dialister and Terrisporobacter are protective factors. This discovery highlights the importance of exploring the underlying mechanisms responsible for these associations. Moving forward, it is crucial for future research to prioritize investigating these mechanisms and developing targeted interventions based on these findings. Our research has shed light on the impact of altered diversity in the gut microbiota on DN, emphasizing the need for further exploration in this area.

## Data availability statement

The dataset analyzed during the current study is publicly available in https://mibiogen.gcc.rug.nl/ and https://gwas.mrcieu.ac.uk/datasets/ebi-a-GCST90018832/.

## Ethics statement

Ethical approval was not required for the study involving humans in accordance with the local legislation and institutional requirements. Written informed consent to participate in this study was not required from the participants or the participants’ legal guardians/next of kin in accordance with the national legislation and the institutional requirements.

## Author contributions

ZY: Data curation, Formal Analysis, Writing – original draft. TS: Formal Analysis, Methodology, Writing – original draft. TZ: Validation, Writing – review & editing. XG: Funding acquisition, Supervision, Validation, Writing – review & editing.
